# *RET*-Targeted Neoadjuvant Therapy Enables Curative Surgery in an Ultra-Elderly Patient With Recurrent Colon Cancer Harboring a CCDC6-RET Fusion

**DOI:** 10.1200/PO-25-01112

**Published:** 2026-04-23

**Authors:** Tianhao Jin, Michele Cricri, Xin Chen, Jinjie He, Yue Liu

**Affiliations:** ^1^ Department of Colorectal Surgery and Oncology (Center for Medical Research and Innovation in Digestive System Tumors, Ministry of Education), The Second Affiliated Hospital of Zhejiang University School of Medicine, Hangzhou, China; ^2^ Ninghai First Hospital, Ningbo, China; ^3^ Department of Clinical Medicine and Surgery, Coloproctology Unit, University of Naples “Federico II”, Naples, Italy

## Introduction

Colorectal cancer (CRC) is among the most common malignancies worldwide, yet the therapeutic landscape remains limited for patients harboring rare oncogenic driver alterations. *RET* fusions lead to constitutive activation of the *RET* kinase, promoting tumor growth and progression.^1,2^ These rearrangements occur in fewer than 1% of CRC cases,^3,4^ defining an aggressive molecular subtype with poor prognosis but marked therapeutic sensitivity to selective RET inhibitors such as selpercatinib and pralsetinib.^5,6^ Although evidence in CRC is still emerging, clinical trials and real-world data have shown that these agents can induce durable responses, particularly in patients progressing after chemotherapy or immune checkpoint inhibitors^7^; however, their application in the neoadjuvant setting has not yet been reported.

We present the case of a 92-year-old woman diagnosed with mucinous adenocarcinoma of the colon harboring a *CCDC6-RET* fusion. The patient experienced early recurrence after surgery and achieved marked tumor regression and radical resection following neoadjuvant treatment with selpercatinib. This case highlights the critical role of comprehensive genomic testing in advanced colorectal cancer.

## Case Presentation

A 92-year-old woman was admitted to out institution with progressive abdominal distension and pain lasting 2 weeks, 8 months after undergoing laparoscopic left hemicolectomy for mucinous adenocarcinoma of the colon.

### Primary Tumor Characteristics

At initial diagnosis, colonoscopy demonstrated a stenosing lesion corresponding to the primary tumor located 38 cm from the anal verge. Baseline tumor markers were elevated (CEA 92 ng/mL; CA19-9 811 U/mL). Presurgery staging was cT4aN2M0. The patient received curative left hemicolectomy. Final pathology showed an ulcerative mucinous adenocarcinoma measuring 10.0 × 5.2 cm, grade 3 differentiation, with tumor infiltration through the serosa, perineural invasion, and vascular emboli. The proximal and distal resection margins were negative (4.0 cm and 4.3 cm from the tumor, respectively). Metastases were detected in four of 13 pericolic lymph nodes and two mesenteric tumor deposits (pT4aN2aM0). Immunohistochemistry showed human epidermal growth factor receptor 2 negativity, high proliferative index (Ki-67 90%), and proficient mismatch repair status (MLH1, MSH2, MSH6, PMS2). Postoperative comprehensive genomic profiling of the primary tumor identified a low-frequency *NRAS* exon 2 mutation (c.35G>A; p.G12D; variant allele frequency 0.62%) and a *CCDC6-RET* fusion (next-generation sequencing performed on an Illumina NextSeq platform). No adjuvant cytotoxic chemotherapy was administrated due to advanced age.

CEA decreased to 24.4 ng/mL, and CA199 decreased to 329 U/mL 5 days after surgery and decreased to normal 3 months later.

### Recurrence and Restaging

**FIG 1.**
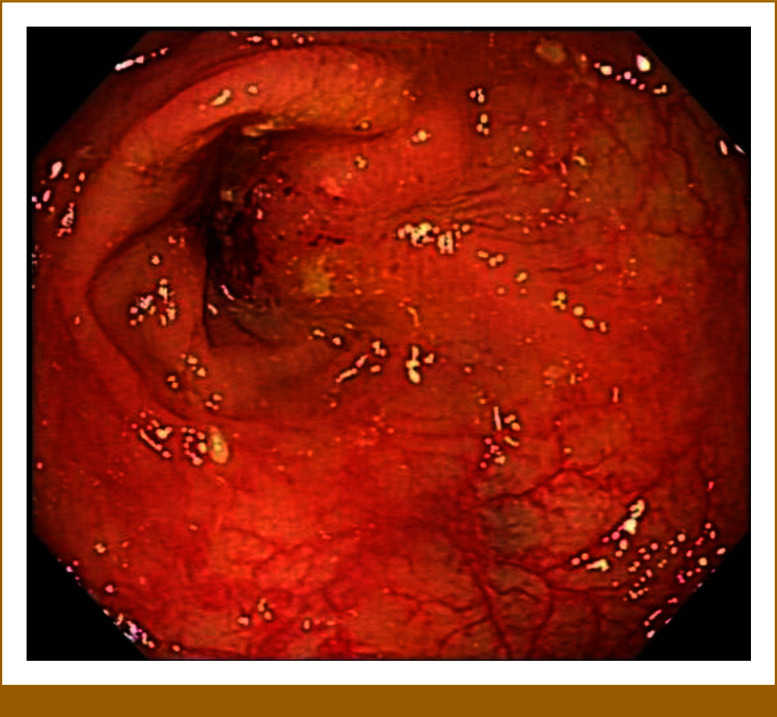
Colonoscopy demonstrating tumor recurrence.

At latest follow-up (8 months after radical surgery), serum tumor markers were markedly elevated (CEA 21 ng/mL; CA19-9 115 U/mL), and the patient developed symptoms of partial bowel obstruction (abdominal bloating after eating solid food). Physical examination indicated a mildly distended abdomen and a palpable mass in the upper quadrant. Contrast-enhanced computed tomography (CT) demonstrated concentric wall thickening and enhancement at the anastomotic site (tumor size 7.5 × 6.1 cm), with indistinct pericolic fat planes and several enlarged regional lymph nodes (maximum diameter 1.5 cm). Colonoscopy showed an ulcerated, stenotic lesion involving approximately three quarters of the luminal circumference (Fig 1); biopsies confirmed mucinous adenocarcinoma. The clinical staging was rT4aN2M0, clinically resectable.

The patient had ECOG PS 1 and preserved organ (cardiac, hepatic, renal) function; however, due to advanced age (92 years) and marked low body reserve (BMI 16.6 kg/m^2^), she was also considered a poor candidate for cytotoxic chemotherapy. Given the short disease-free survival (DFS) time after the first surgery, with the presence of an actionable *RET* fusion, the multidisciplinary team (MDT) recommended a short-course targeted therapy with selpercatinib (160 mg twice daily), which may improve the DFS and quality of life for this patient.

### Treatment Response and Clinical Course

Selpercatinib was well tolerated, with only mild leukopenia (lowest WBC count 3.1 × 10^9^/L) and transient dizziness, which was managed conservatively. After 2 weeks of treatment, serum markers declined sharply: CEA decreased from 21 to 8.9 ng/mL and CA19-9 from 115 to 28.9 U/mL.

**FIG 2.**
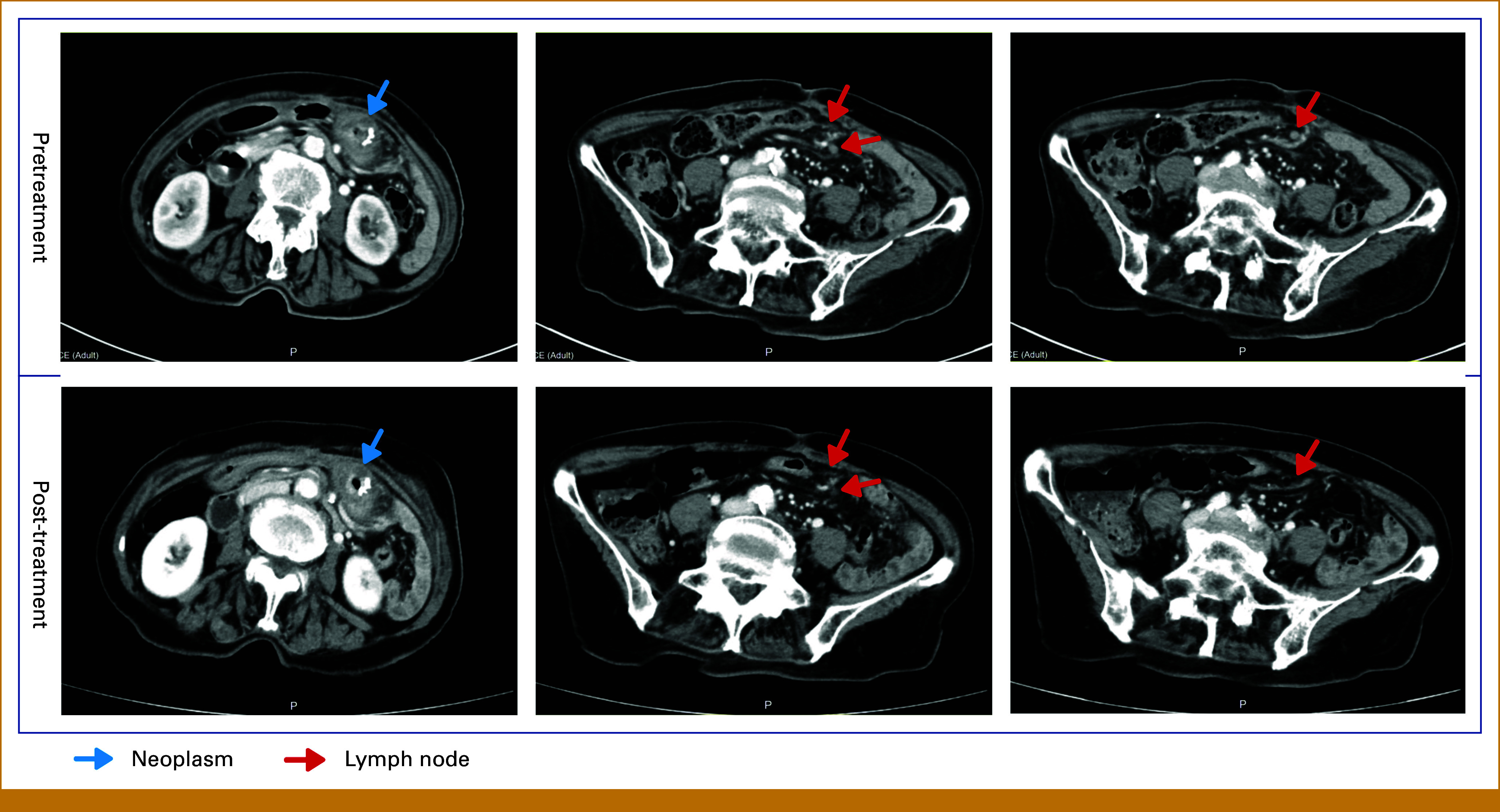
Computed tomography before and after 2 weeks of treatment with selpercatinib.

Contrast-enhanced CT demonstrated a reduction in the anastomotic recurrence from 7.5 × 6.1 cm to 5.0 × 4.5 cm, with concomitant decrease in regional lymphadenopathy (largest node from 1.5 cm to 0.5 cm in maximum diameter; Fig 2), accompanied by complete resolution of abdominal distension and obstructive symptoms. Given the remarkable biochemical and radiologic response, and her improved clinical condition of obstruction, the patient underwent completion transverse colon resection with 1-week discontinuation of selpercatinib.

**FIG 3.**
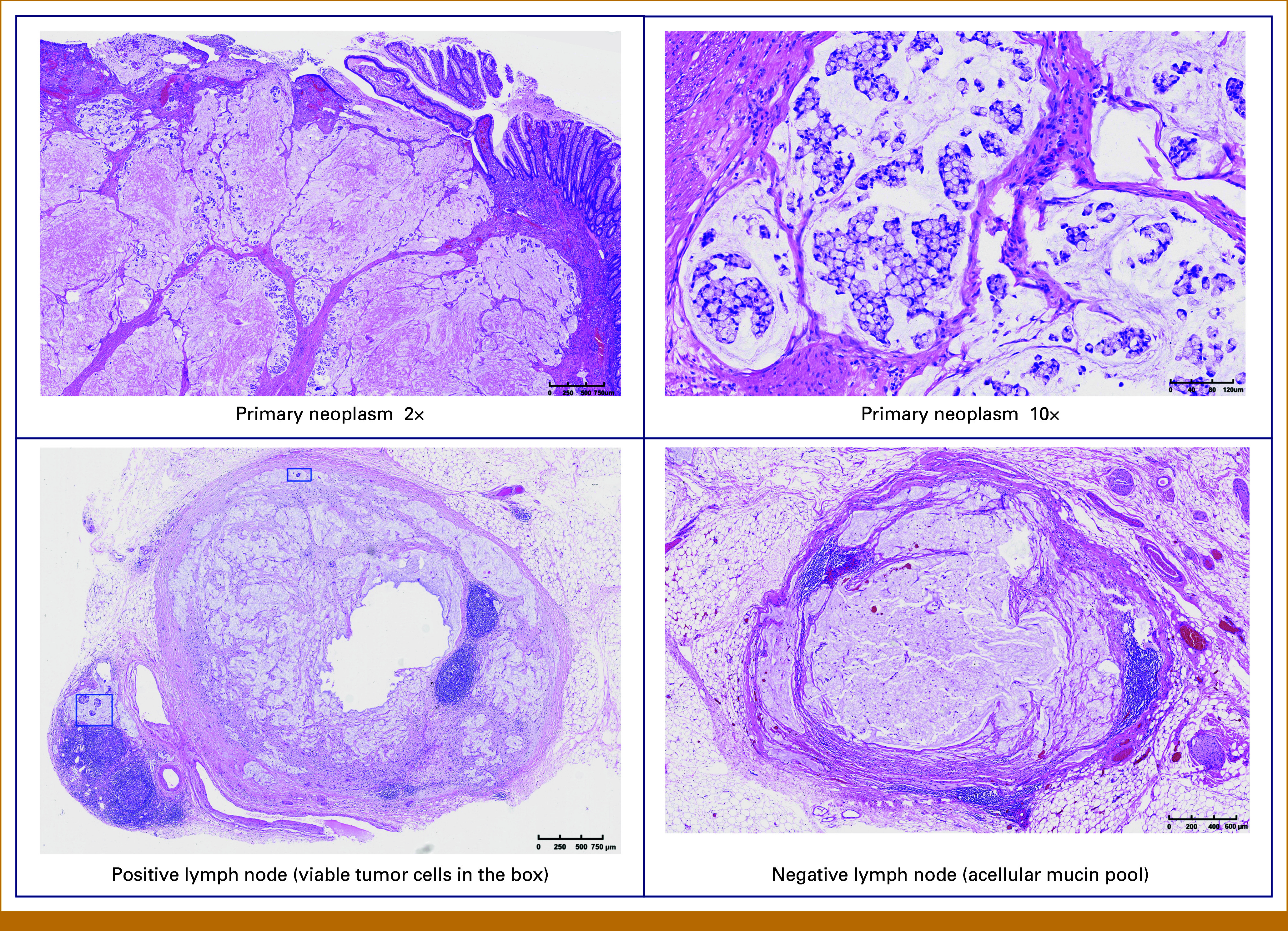
Pathology evaluation of primary neoplasm and lymph node after surgery.

Histopathologic examination revealed residual adenocarcinoma with evidence of tumor regression (ypT3N1bM0, tumor regression grade [TRG] 2). Histopathology after surgery indicated mucinous adenocarcinoma, with partial signet ring carcinoma. The tumor size measured 5 × 5 × 1.6 cm, the proximal and distal tumor margins are negative, and lymphovascular invasion existed. Sixteen lymph nodes were resected, and three were tumor cell–positive. Mucus lake was seen in 13 lymph nodes without tumor cells (Fig 3).

After surgery, the CEA and CA199 both decreased to normal.

### Outcome and Follow-Up

Postoperative recovery was uneventful. Pathology demonstrated residual viable tumor (no pathologic complete response; TRG 2). Adjuvant selpercatinib was resumed 1 month after surgery and was continued for 1 month without clinically significant toxicity.

**FIG 4.**
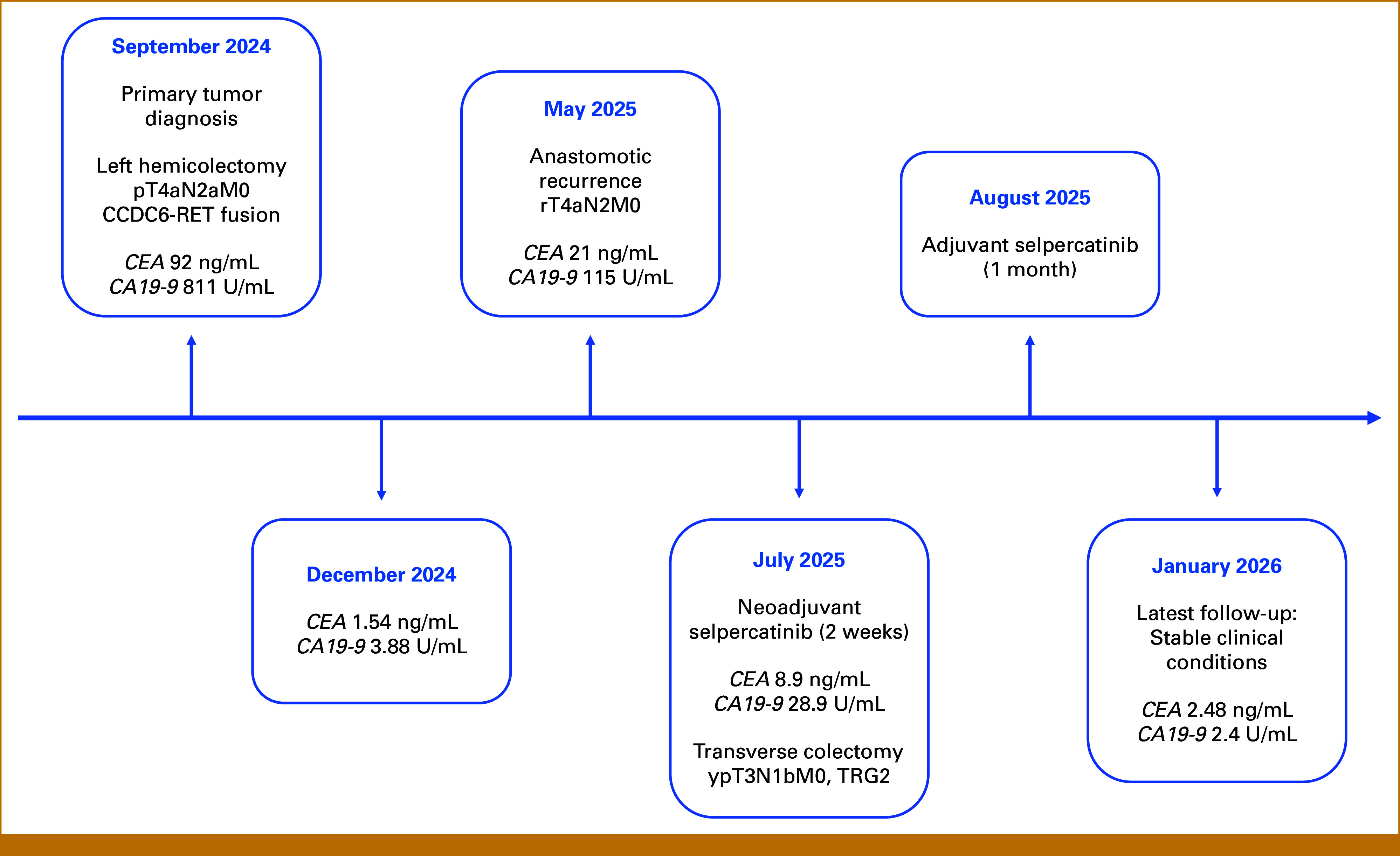
Timeline of the patient's clinical journal.

During the latest examination (7 months after surgery), CEA 2 was 0.48 ng/mL and CA199 2.40 U/mL (Fig 4).

Written informed consent was obtained from the patient for publication of this case and accompanying images.

## Discussion

The management of CRC in ultra-elderly patients poses major clinical challenges, as comorbidities and frailty frequently preclude the use of standard cytotoxic regimens. Nonetheless, the growing availability of targeted and biomarker-driven therapies offers new opportunities for individualized management, even in patients traditionally considered ineligible for active treatment. In this case, a multidisciplinary approach integrating molecular diagnostics and clinical judgment enabled the use of selpercatinib in a 92-year-old woman with recurrent mucinous adenocarcinoma harboring a *CCDC6-RET* fusion, ultimately allowing curative-intent resection after rapid tumor regression.

### Precision Medicine and Multidisciplinary Decision Making in the Ultra-Elderly

Although the incidence of CRC increases with age, treatment decisions in patients over 80 years remain complex.^8^ Elderly individuals often exhibit decreased physiologic reserve and increased risk of toxicity from chemotherapy, which frequently limits therapeutic intensity and compromises outcomes. Several studies have demonstrated that fit elderly patients may still benefit from systemic therapy, but real-world data confirm a consistent trend toward undertreatment.^9,10^ In this context, MDT evaluation becomes essential to tailor interventions to biological rather than chronological age. In our case, the patient had early postoperative recurrence after left hemicolectomy but was deemed unsuitable for conventional chemotherapy due to advanced age and frailty. Genomic profiling revealed a rare *CCDC6-RET* fusion, allowing the MDT to adopt a precision-based strategy with selpercatinib—a highly selective RET inhibitor with favorable safety and tolerability. This approach achieved substantial tumor downstaging within 2 weeks, transforming a palliative scenario into a surgical opportunity. The case underscores that precision medicine, when guided by multidisciplinary discussion, can extend curative options even to very old patients who would otherwise receive only supportive care.

### RET Fusion–Positive CRC as a Distinct Molecular Subtype

RET gene rearrangements occur in <1% of CRC cases, but they define a distinct molecular entity associated with aggressive clinical behavior. *RET* fusion–positive tumors often exhibit high Ki-67 proliferation indices, poor differentiation, and vascular invasion, as seen in our patient. Prior analyses have reported shorter overall survival and poor responses to standard chemotherapy compared with *RET*-negative CRC, emphasizing the need for alternative targeted approaches.^7^

For our patient, the presence of a *CCDC6-RET* rearrangement aligned with the aggressive clinical course—early relapse within 8 months after surgery and rapid local progression with multiple lymph node metastases.

### Emerging Neoadjuvant Potential of Selective RET Inhibitors

Selective *RET* inhibitors, such as selpercatinib and pralsetinib, have shown remarkable clinical activity across multiple *RET* fusion–positive solid tumors, including non–small cell lung cancer and thyroid carcinoma. In colorectal cancer, their use has been limited to advanced or metastatic settings, where response rates of 20%-30% and excellent safety profiles have been reported.^11^ However, evidence regarding neoadjuvant use remains anecdotal. In this case, selpercatinib was used as short-course neoadjuvant therapy, achieving rapid biochemical and radiologic response with minimal toxicity, followed by successful R0 resection. Postoperative pathology confirmed partial histologic regression (TRG 2), supporting biological activity of *RET* inhibition in an *RET* fusion–positive colorectal cancer in this chemotherapy-naïve patient. This experience highlights the potential role of RET inhibitors as part of a multimodal strategy in selected patients with locally recurrent or borderline resectable disease, particularly when standard cytotoxic therapy is not feasible. Prospective studies are warranted to evaluate optimal duration, timing, and integration of *RET* inhibitors in preoperative settings.

## Conclusion

This case represents, to our knowledge, the first reported instance of neoadjuvant RET inhibition enabling curative resection in colorectal cancer. It also demonstrates that comprehensive molecular profiling can uncover actionable alterations even in ultra-elderly patients with recurrent colorectal cancer. Identification of a *CCDC6-RET *fusion enabled a precision-based strategy with selpercatinib, achieving rapid tumor regression and allowing curative resection despite advanced age. The favorable safety profile and marked response observed suggest that selective *RET* inhibition may have a role beyond metastatic settings, including neoadjuvant applications in selected patients. Broader implementation of genomic testing and integration of multidisciplinary decision making are essential to extend precision oncology benefits to all patients with CRC, regardless of age or comorbidities.
